# Less blood loss in supercapsular percutaneously assisted versus posterolateral total hip arthroplasty

**DOI:** 10.1186/s13018-021-02363-z

**Published:** 2021-03-25

**Authors:** Yong Hu, Min-Cong Wang, Tao Wang, Yue Meng, Xiao-Min Chao, Hui-Feng Zhu, Cheng-Guo Li, Cheng-Long Pan, He-Bei He

**Affiliations:** 1 Present Address: Department of Joint Surgery, The Fifth Affiliated Hospital of Southern Medical University, Guangzhou City, Guangdong Province China; 2 Present Address: Department of Orthopaedic Surgery, The Third Affiliated Hospital of GuangZhou Medical College, Guangzhou City, Guangdong Province China

**Keywords:** Total hip arthroplasty, Supercapsular percutaneously assisted, Minimally invasive, Blood loss, Transfusion

## Abstract

**Background:**

Although excellent clinical outcomes of supercapsular percutaneously assisted total hip arthroplasty (SuperPath) have been reported, the peri-operative blood loss has rarely been reported. The current study determined the blood loss during SuperPath and compared the blood loss with conventional posterolateral total hip arthroplasty (PLTH).

**Methods:**

This retrospective study enrolled patients who underwent unilateral primary THA between January 2017 and December 2019. The demographic data, diagnoses, affected side, radiographic findings, hemoglobin concentration, hematocrit, operative time, transfusion requirements, and intra-operative blood loss were recorded. The peri-operative blood loss was calculated using the OSTHEO formula. Blood loss on the 1st, 3rd, and 5th post-operative days was calculated. Hidden blood loss (HBL) was determined by subtracting the intra-operative blood loss from the total blood loss.

**Results:**

Two hundred sixty-three patients were included in the study, 85 of whom were in the SuperPath group and 178 in the posterolateral total hip arthroplasty (PLTH) group. Patient demographics, diagnoses, affected side, operative times, and pre-operative hemoglobin concentrations did not differ significantly between the two groups (all *P* > 0.05). Compared to the PLTH group, the SuperPath group had less blood loss, including intra-operative blood loss, 1st, 3rd, and 5th post-operative days blood loss, and HBL (all *P* < 0.05). Total blood loss and HBL was 790.07 ± 233.37 and 560.67 ± 195.54 mL for the SuperPath group, respectively, and 1141.26 ± 482.52 and 783.45 ± 379.24 mL for the PLTH group. PLTH led to a greater reduction in the post-operative hematocrit than SuperPath (*P* < 0.001). A much lower transfusion rate (*P* = 0.028) and transfusion volume (*P* = 0.019) was also noted in the SuperPath group.

**Conclusion:**

SuperPath resulted in less perioperative blood loss and a lower transfusion rate than conventional PLTH.

## Introduction

Total hip arthroplasty (THA) is an effective treatment option for advanced degenerative and ischemic hip arthropathy. The traditional posterolateral approach is the most widely applied approach for primary and revision hip arthroplasties [1]. Despite the advantages of good exposure to the hip capsule and easing the insertion of implants, the considerable peri-operative blood loss during posterolateral total hip arthroplasty (PLTH) approach has hindered popularization of this approach [2]. Hidden blood loss (HBL), possibly associated with post-operative bleeding from muscle, tendon, and bone trauma, has been reported and has attracted attention, which accounts for 24–60% of the total blood loss [3, 4]. Minimally invasive THA has been developed with the rationale that less tissue trauma reduces the surgical blood loss and hastens patient recovery [5].

The fact that the length of incision is less important with respect to surgical outcome than disruption of muscles, impairment of soft tissue vascularization, innervation, and preservation of bone has been increasingly accepted by surgeons [6]. A novel minimally invasive surgical technique and initial experience with supercapsular percutaneously assisted total hip arthroplasty (SuperPath) was introduced by Dr. James Chow in 2011 [7]. SuperPath uses the interval between the gluteus minimus and piriformis, accesses the capsule from the top, prepares the femur without dislocating the femoral head, and reams the acetabulum through an accessory portal, which preserves capsular attachments and maintains the integrity of the external rotators [8]. This tissue-sparing technique is advocated with reported advantages of a low complication rate, excellent gait kinematics, a low transfusion rate, and a shortened length of hospital stay [9]. Compared to conventional PLTH, SuperPath allows for early post-operative rehabilitation and a faster recovery [10].

Existing studies mainly have emphasized the clinical outcomes of the SuperPath approach [10–12]. To our knowledge, the existing study that reported the peri-operative blood loss of SuperPath was limited. Surgical blood loss is always a concern among orthopedic surgeons because of the close relationship with allogenic blood transfusion. A large demand for transfusion has been reported due to the considerable perioperative blood loss during THA. Indeed, a previous study reported that 16–37% of patients who underwent THA needed a blood transfusion [13]. Accordingly, the risk of transfusion complications is increased, such as infections and immunologic reactions [14]. Therefore, we determined the peri-operative blood loss in SuperPath, and compared blood loss with conventional PLTH in the current study. We hypothesized that SuperPath resulted in less blood loss due to a mini-incision and preservation of the external rotators, hip capsule, and abductor integrity.

## Methods

### Patients

This retrospective study enrolled patients who underwent unilateral primary THA between January 2017 and December 2019. The surgical technique used SuperPath or PLTH. The common exclusion criteria were as follows: active infection; tumors; and hematologic diseases, such as blood coagulation disorders, thrombocytopenia, and other hemorrhagic disorders. The additional exclusion criteria for SuperPath were as follows: severe deformation of the proximal femur; congenital high hip dysplasia; and osteosynthesis of the proximal femur [15]. Variables, such as gender, age, height, weight, BMI, diagnoses, affected side, pre- and post-operative radiographic findings, pre-operative hemoglobin (Hb) concentrations, pre- and post-operative hematocrit (Hct), operative time, and transfusion blood volume were recorded.

### Surgical protocol

Preoperative preparations were the same between the two groups, such as general condition assessment, patient educations, carbohydrate loading, and preoperative digital templating measurements. The surgery was performed under general or continuous epidural anesthesia. Antibiotics were routinely administered intra-operatively and 24 h post-operatively. One gram of tranexamic acid was administered intravenously at the beginning of the operation. Periarticular injection of analgesics consisting of 7.5 mg of ropivacaine, 7.5 mg of adrenaline, and 10 mg of betamethasone was performed, as suggested by Pepper et al. [16]. One gram of tranexamic acid was topically infiltrated after closing incision. All surgeries were performed by the same senior orthopedic chief surgeon. All patients received cementless THA implants. Drainage was not used after incision closure to obtain enhanced recovery after surgery [17].

#### SuperPath approach

The patient was placed in the standard lateral decubitus position with the involved leg in the “home position,” (45°–60° of flexion, 20°–30° of internal rotation) [18]. An incision was made from the tip of the greater trochanter 6–8 cm proximally in line with the femur. The gluteus maximus muscle was split by blunt dissection, followed by the gluteus medius and minimus, and the piriformis tendon was retracted to access the capsule without dissecting any muscles. The capsule was incised along the path of the skin incision from the saddle of the femoral neck to 1 cm proximal to the acetabular rim. A sharp starter reamer was used to create the femoral canal, then sequential femoral broaches were used to complete the preparation and size the proximal femoral canal (MicroPort Scientific Corporation, Shanghai, China). The femoral neck osteotomy was performed level with the broach neck. Acetabular preparation and cup impaction were completed through a portal without needing release of the iliotibial band or remaining external rotators [18]. The reamer shaft was passed through the cannula and mated with the acetabular reamers inside the capsule. Following sequential reaming to size, the chosen acetabular implant was placed through the main incision. The final components were implanted after satisfactory trial reductions. Finally, closure was limited to the capsule, fat, and skin because no muscles were dissected during this procedure. The capsule was anatomically repaired with a running suture or single stitches.

#### Posterior-lateral THA approach

The patient was placed in a lateral position. The incision was made 6–8 cm anterior to the posterior superior iliac spine and distal to the iliac crest, overlying the anterior border of the gluteus maximus muscle, then extended distally to the anterior edge of the greater trochanter and further distally along the line of the femur for 12–15 cm. The iliotibial band was cut in line with its fibers, extending proximally to the greater trochanter. A further incision was performed to expose the greater trochanter and the muscles that insert into the greater trochanter. Blunt dissection was used to separate the posterior border of the gluteus medius muscle from the adjacent piriformis tendon. After identification of the piriformis, the short external rotators and piriformis were dissected at their insertion, then tagged with a braided suture for identification and repaired at the end of the procedure. The capsule was incised superiorly in the axis of the femoral neck from the acetabulum to the intertrochanteric line. The posterior dislocation of the hip joint was completed by flexion of the hip, and medial adduction and internal rotation of the lower limb. The standard posterior technique was as follows: femoral neck osteotomy, acetabulum and femoral preparations, and prosthesis implantation. Repair of the posterior capsule and reattachment of the external rotators were competed before closure of the skin incision.

### Post-operative treatment

**Fig. 1:**
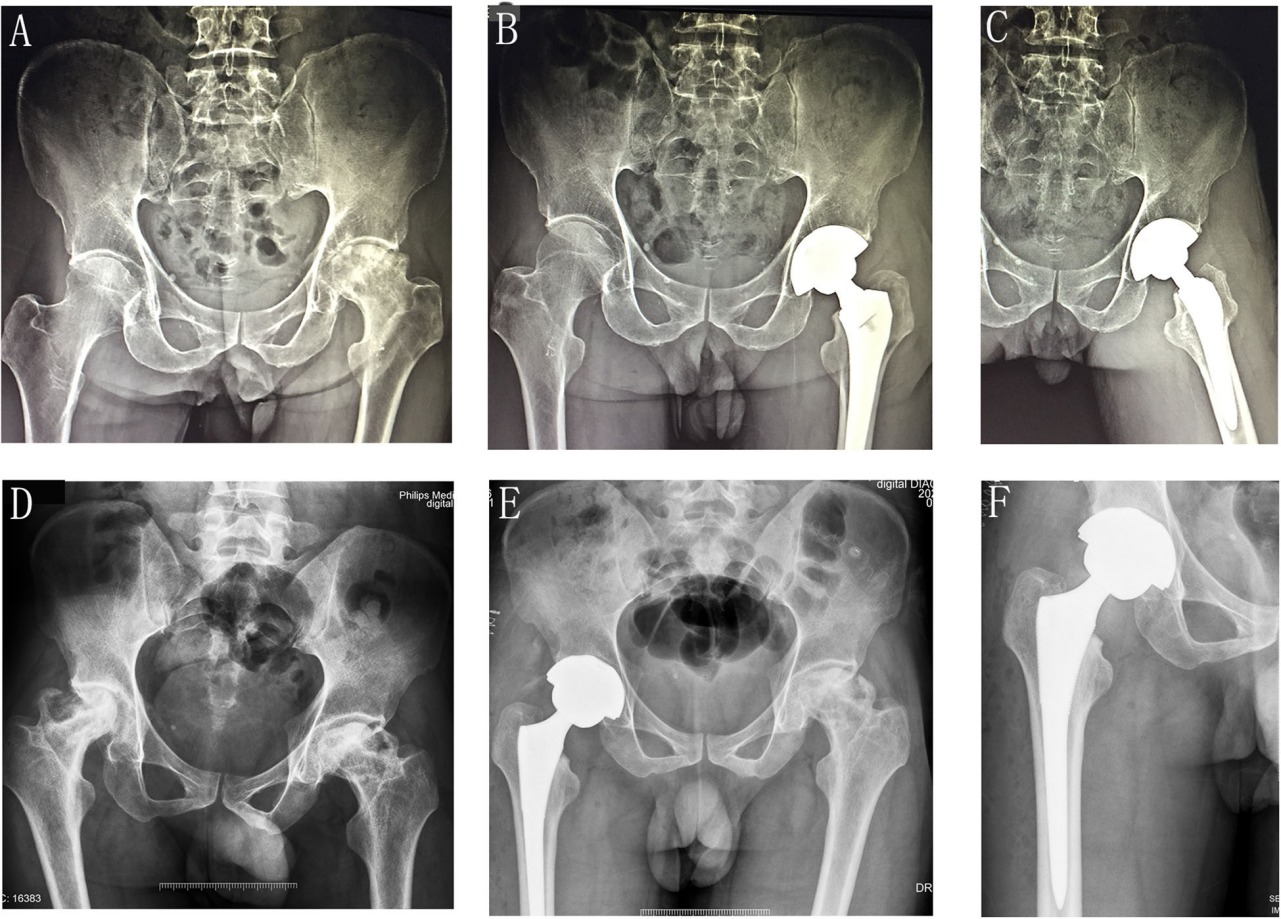
**a**–**c** Radiographic for patients undergoing SuperPath (**a** preoperative image for a necrosis of femoral head, left hip; **b**, **c** postoperative image). **d**–**f** Radiographic for patients undergoing PLTH (**d** preoperative image for a necrosis of femoral head, right hip; **e**, **f** postoperative image)

All patients received the same rehabilitation protocol by a physical therapist in our hospital. Patients can begin indoor walking independently using crutches when tolerating weight-bearing under the supervision of physical therapists. Combined venous thrombosis embolism prophylaxis with mechanical and pharmacologic methods was provided during hospitalization. Blood parameters were routinely tested on the 1st, 3rd, and 5th post-operative days. Post-operative radiographs were obtained to evaluate the place of implants (Fig. 1).

### Estimation of blood loss

The intra-operative blood loss was estimated by weighing sterile cotton gauze pads and analysis of the aspirated blood volume. Considering the significant difference between estimated and calculated blood loss, the latter was the main method of evaluation [19]. Peri-operative blood loss was calculated using the Orthopedic Surgery Transfusion Hemoglobin European Overview (OSTHEO) formula [19, 20], which was based on the change in Hct. The estimated blood loss on the 1st, 3rd, and 5th post-operative days was included. The detailed formulae are shown in Table 1.

**Table 1:** Estimations of Perioperative Blood Loss Volume

**Orthopedic Surgery Transfusion Hemoglobin European Overview (OSTHEO) formula**: ① Estimated blood volume (EBV) = (0.0235 × height in cm^0.42246 × weight in kg^0.51456) × *k*, where *k* = 2430 for women and 2530 for men. ② Total RBC loss (mL) = [Uncompensated RBC loss (mL) + Compensated RBC loss (mL)]/0.35 Uncompensated RBC loss (mL) = EBV × (preoperative Hct levels − postoperative Hct levels), Compensated RBC loss (mL) = allogeneic erythrocyte units × 150 + autologous transfusion volume × 0.3. Notably, postoperative Hct levels were collected from the laboratory test which was carried out on the 1st, 3rd, and 5th day after operation. Only transfusions that were executed before phlebotomizing for laboratory test were included in the study.	

### Transfusion protocol

Autogenous blood was not applicable in the current study. Allogenic transfusion was performed based on the British guidelines (22) and clinical judgment. The traditional view on transfusion triggers was a Hb < 70 g/L and a hematocrit < 25%. A Hb < 80 g/L was a transfusion trigger for patients with cardiovascular and respiratory disorders or patients > 65 years of age. Acute anemia, a drop in blood pressure (< 90/60 mmHg), dizziness, lip pallor, weakness, and shortness of breath were also transfusion triggers.

### Statistical methods

All statistical analyses were performed using SPSS for Windows (version 23.0; SPSS Inc., Chicago, IL, USA). Continuous data are presented as the mean ± standard deviation (SD). Comparisons of quantitative variables were performed using an unpaired Student’s *t* test. In the case of heteroscedasticity, the Mann-Whitney *U* test was used. Repeated measure analysis of variance was used to compare the change in Hct between the two groups. A chi-square test was executed to compare the frequencies of qualitative variables. Differences at a *P* < 0.05 level were identified as statistically significant.

## Results

A total of 263 patients who had complete records were included in the study. Eighty-five patients (17 males and 68 females) with a mean age of 67 years (range 23–84 years) underwent SuperPath. In the PLTH group, there were 178 patients (25 males and 153 females) with a mean age of 65 years (range 28–82 years). Demographic data and diagnoses were summarized in Table 2. There were no significant differences in age, gender, BMI, diagnoses, and involved side between the SuperPath and the PLTH groups (all *P* > 0.05). In the SuperPATH group, the diagnoses included femoral neck fracture (FNF; 23.5%), necrosis of femoral head (ONFH; 63.5%), and hip osteoarthritis (OA; 13%). In the PLTH group, the diagnoses included FNF (24.2%), ONFH (52.2%), and OA (23.6%).

**Table 2:** Demographics and diagnosis of the patients

		SuperPath group	PLTH group	*P*
Number of patients		85	178	
Age (years)		66.91 ± 9.14	65.22 ± 8.69	0.149
BMI (kg/m^2^)		25.98 ± 3.13	26.47 ± 4.07	0.577
Gender	Male	17	25	0.280
	Female	68	153	
Diagnosis	FNF	20	43	0.102
	ONFH	54	93	
	OA	11	42	
Involved hip	Left	34	86	0.234
	Right	51	92	

### Blood loss and transfusion

**Fig. 2:**
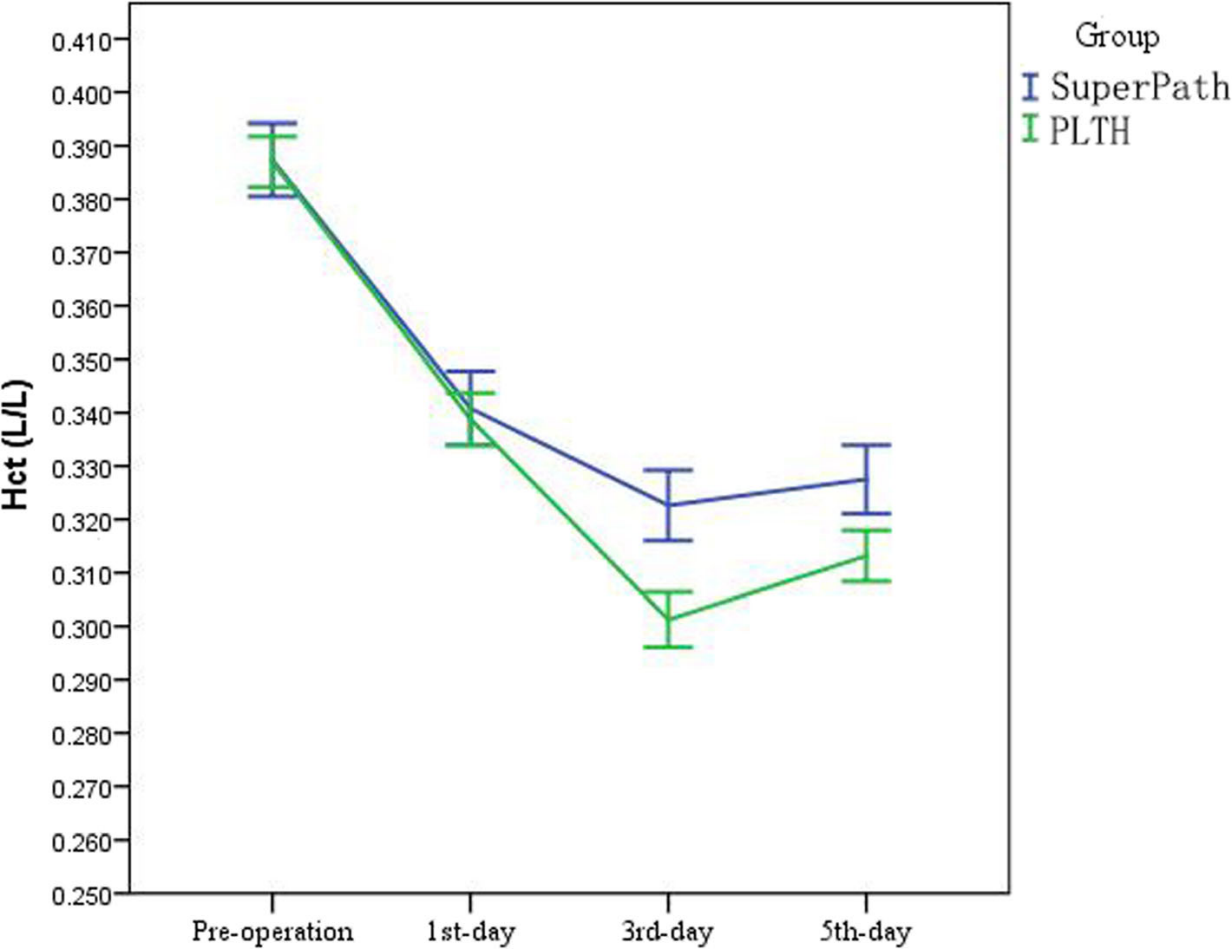
Hct was decreased postoperatively in both groups. PLTH resulted in a greater reduction in the Hct post-operatively than SuperPath (*P* < 0.001)

Operative time and the pre-operative Hb concentration were not statistically different between the two groups (*P* = 0.111 and 0.871, respectively). An apparent decrease in the Hct was noted post-operatively in both groups. PLTH resulted in a greater reduction in the Hct post-operatively than SuperPath (*P* < 0.001; Fig. 2). Patients in the SuperPath group had less intra-operative blood loss (*P* < 0.001; 95% CI, − 154.62 to − 102.17), and day 1 (*P* = 0.036; 95% CI, − 324.58 to − 178.03), day 3 (*P* < 0.001; 95% CI, − 549.55 to − 373.51), and day 5 post-operatively (*P* < 0.001; 95%CI, − 467.70 to − 277.83) blood loss than the PLTH group. The surgical blood loss reached a maximum on day 3 post-operatively in the SuperPath and PLTH groups. Thus, this volume was considered as the total blood loss (TBL), which peaked at 790.07 ± 233.37 mL in the SuperPath group and 1141.26 ± 482.52 mL in the PLTH group. The hidden blood loss (HBL) was estimated by subtracting the intra-operative loss from the TBL [21]. As a result, the HBL was 560.67 ± 195.54 mL in the SuperPath group, and 783.45 ± 379.24 mL in the PLTH group (*P* < 0.001; 95% CI, − 292.63 to − 152.95).

**Fig. 3:**
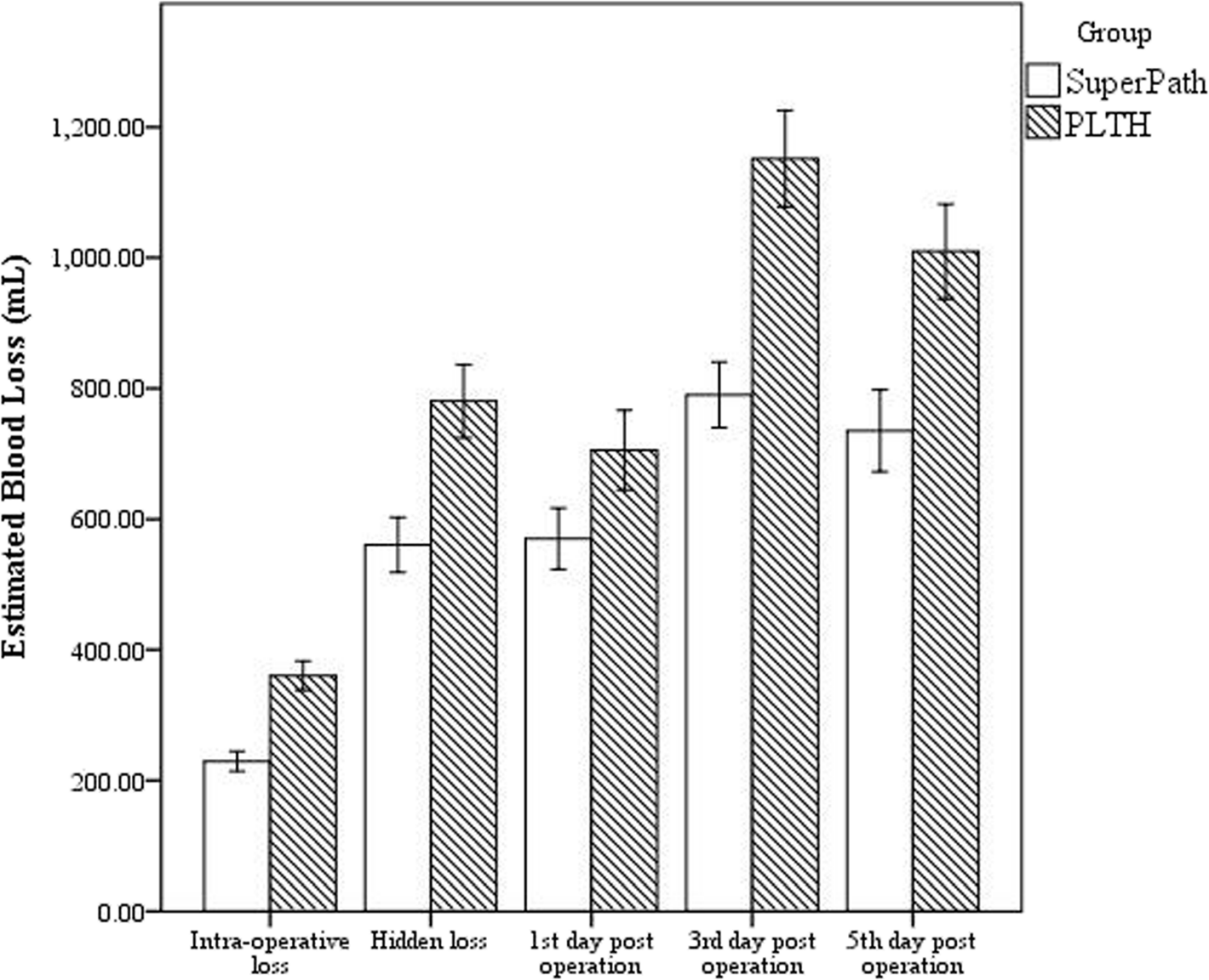
Much less perioperative blood loss was noted in SuperPath, including intra-operative blood loss, hidden blood loss, day 1, day 3, and day 5 post-operative blood loss (all *P* < 0.05)

The SuperPath group had a significantly lower transfusion rate than the PLTH group (5.8% and 15.7%, respectively; *P* = 0.028). Accordingly, a lower allogenic blood volume was required in the SuperPath group than the PLTH group (*P* = 0.019; 95% CI, − 0.355 to − 0.123). The detailed results were presented in Table 3 and Fig. 3.

**Table 3:** Patients’ perioperative data

	SuperPath group	PLTH group	*P* values
Preoperative hemoglobin (g/L)	127.27 ± 12.52	127.98 ± 12.07	0.638
Preoperative hematocrit (L/L)	0.387 ± 0.018	0.387 ± 0.324	0.916
Operation time	100.72 ± 12.18	98.34 ± 11.04	0.111
Intra-operative blood loss (mL)	229.41 ± 70.64	357.81 ± 145.30	< 0.001
Blood loss (1st day) (mL)	570.26 ± 218.27	699.03 ± 482.53	0.036
Blood loss (3rd day) (mL)	790.07 ± 233.37	1141.26 ± 482.52	< 0.001
Blood loss (5th day) (mL)	735.10 ± 291.91	1002.20 ± 483.53	< 0.001
Hidden blood loss (mL)	560.67 ± 195.54	783.45 ± 379.24	< 0.001
Transfusion rate	5.8% (5/85)	15.7% (28/178)	0.028
Transfusion volume (u)	0.060 ± 0.237	0.300 ± 0710	0.019

## Discussion

In the current study, we focused on the surgical blood loss associated with the SuperPath approach and compared to the PLTH approach. In agreement with Sehat et al. [21] and Shen et al. [22], we found that the surgical blood loss reached a maximum on day 3 post-operatively in the SuperPath and PLTH groups. Thus, this volume was defined as the TBL, which was 1141 mL for patients who underwent PLTH in the current study. Miao et al. [4] enrolled 322 patients who underwent PLTH and reported that the TBL was 1155 ± 377 mL, which was in agreement with our findings. Another study by Shen et al. [22] reported a larger amount of TBL for PLTH (1438 mL). For patients in the SuperPath group, the TBL was much less (790.07 ± 233.37 mL). Greater than 300 mL TBL occurred in the PLTH group than the SuperPath group. Accordingly, PLTH also resulted in a greater reduction in Hct post-operatively than SuperPath.

Meng et al. [1] compared the short-term outcomes of staged THA with SuperPath or through the posterolateral approach for bilateral ONFH in a prospective study; however, their findings were opposite to our results. Meng et al. [1] reported that the mean blood loss was higher in the SuperPath group (1108.50 mL) than the PLTH group (843.50 mL). Of note, only four male patients were enrolled in Meng et al. [1] study. Thus, the limited sample size may compromise the conclusions. Meng et al. [1] argued that the unexpected outcomes were possibly attributed to the intra-operative mechanical stresses from the specific trocar cannula and the elongated operative times during SuperPath. Our findings were also inconsistent with their results, which showed comparable operation time between the SuperPath and PLTH groups. The learning curve for SuperPath is thought to account for the discrepancy [23]. Another study conducted by Xie et al. [10] suggested that the intra-operative blood was comparable between SuperPath and PLTH, which was also inconsistent with our results. Xie et al. [10] attached importance to the use of electrocautery when releasing external rotators close to the bone surface, which might not lead to a large amount of blood loss during the conventional posterolateral approach.

We also favored the hemostatic effect of electrocautery in THA; however, our findings indicated that the increased blood loss during the PLTH approach was mainly attributed to dissecting the piriformis, obturator, gemellus superior, and gemellus inferior, in agreement with Chimento et al. [24] and Mazoochian et al. [25]. Repantis et al. [26] proposed that the main source of blood loss is the bone (femur and acetabulum) in a cementless THA. Thus, Repantis et al. [26] found that no significant differences in perioperative blood loss were present between the minimally invasive approach and the conventional approach that completely released gluteus minimus insertion, partially released gluteus medius insertion, and subtotally, resected capsule. Our findings suggested that the intra- and post-operative blood loss caused by impairment of soft tissues cannot be omitted.

Although SuperPath has been advocated as a “micro” (external rotator sparing) and “mini” (external rotator sacrificing) posterior approach [7], the significant post-operative HBL (560.67 mL) cannot be dismissed, which on average accounted for 70.96% of the TBL. We reported more HBL in the PLTH group compared to the SuperPath group, which reached 783.45 mL (accounting for 68.64% of the TBL). The large amount of HBL might attribute to lacking intravenous administration of tranexamic acid post operation. Administration of tranexamic acid is an effective strategy when compared to placebo for reducing calculated blood loss and the need for transfusion during the perioperative episode of a primary THA; however, its optimal administration remains controversia l[27]. Fibrinolysis activation contributes to post-operative bleeding after THA, which reaches peak 6 h after THA and then gradually decreases to the pre-operative level 24 h after the operation [28, 29]. Jia [28] found that the serum antifibrinolytic activity of IV tranexamic acid with one dose merely maintained for 7 to 8 h; thus, another dose of tranexamic acid post operation is favored to reduce HBL.

We believed that perseveration of integrity of muscle without impairing any muscular artery/vein should mainly contribute to a reduction of post-operative bleeding for SuperPath. During the operation, the gluteus maximus muscle was split by blunt dissection in line with the fibers, followed by the gluteus medius and minimus, and the piriformis tendon were retracted to access to the capsule without dissecting any muscles [11].

The entire capsular incision is perfectly repaired with a continuous suture after implantation during SuperPat h[18]. Hip capsule preservation is a revolutionary concept that ensures anatomic restoration, length, and offsets near the native joint [8]. The pressure after anatomically repairing the capsule may exert a hemostasis effect by compression. Liu et al. [3] proposed that retaining and repairing the articular capsule cannot only significantly decrease the hidden blood loss, but also the peri-operative bleeding. Furthermore, preservation of the short external rotators and posterior capsular allowed no particular range of motion restrictions post-operatively and achieved a high level of function with a very low dislocation risk. The dislocation rate of SuperPath (0.8%) was significantly decreased from those reported in some recent THA studies (2.9–6.0%) [11]. Bergin et al. [30] suggested that the tissue-sparing minimally invasive approach resulted in less surgical trauma and possibly lower levels of inflammation. The inflammation cascade will inevitably impair the tissue through inducing cytokines, lysosomal enzymes, free oxygen radicals, and metabolic derivatives of arachidonic acid, which leads to endothelial cell injury and increased bleeding. The inflammation cascade might also be responsible for the destruction of erythrocytes because hemolysis is considered an important reason for HBL [31, 32].

A large TBL can result in the need for an allogenic transfusion. In the current study, we noted a significantly higher transfusion rate in the PLTH group compared to the SuperPath group. The transfusion rate was 5.8% for SuperPath, whereas it reached 15.7% for PLTH. A multicenter, retrospective study by Gofton et al. [11] also reported a low transfusion rate for patients who underwent SuperPath (3.3%). In contrast, Yoshihara et al. [33] reported that the overall transfusion rate for THA was 25.5%. Another study by Mednick et al. [34] reviewed 27130 primary THAs and found a transfusion rate of 22.2%. Allogenic transfusion is associated with several severe complications, such as hemolytic reactions, graft versus host disease, transfusion-associated circulatory overload, and transfusion-related acute lung injury. Gofton et al. [11] reported that the 30-day all-cause readmission rate was 2.3 % or a 1.9% reduction from the described national average for SuperPath, profiting by the low transfusion and complication rates.

There were several limitations in our study. First, the present study was a retrospective case series. In addition, the significant difference in gender ratio was observed in the enrolled patients. More female patients were enrolled than male patients. The sample size was relatively small, which might have influenced the power of significance. A prospective, randomized controlled trial is warranted to more appropriately evaluate the peri-operative blood loss of different approaches.

## Conclusion

In our study, we demonstrated that SuperPath is a veritable minimally invasive approach that results in less intra-operative blood loss, TBL, and HBL than the conventional PLTH approach, which was consistent with our initial hypothesis. On average, the TBL (300 mL) was less in the SuperPath group than that in the PLTH group. A much lower transfusion rate was also noted in the SuperPath group (5.8% vs. 15.7%). Therefore, our results indicated that the SuperPath approach is a reliable technique for patients who need a primary THA.

## Data Availability

The datasets used and/or analyzed during the current study are available from the corresponding author on reasonable request.

## Abbreviations

THA: Total hip arthroplasty
PLTH: Posterolateral total hip arthroplasty
SuperPath: Supercapsular percutaneously assisted total hip arthroplasty
HBL: Hidden blood loss
TBL: Total blood loss
Hb: Hemoglobin
Hct: Hematocrit
OSTHEO: Orthopedic Surgery Transfusion Hemoglobin European Overview
